# Effect of eliminating open defecation on diarrhoeal morbidity: an ecological study of Nyando and Nambale sub-counties, Kenya

**DOI:** 10.1186/s12889-016-3421-2

**Published:** 2016-08-04

**Authors:** John Njuguna

**Affiliations:** Mukurwe-ini sub-County Public Health Office, P.O. Box 112-10103, Mukurwe-ini, Kenya

**Keywords:** Diarrhoea, Open defecation, Kenya, Community- led total sanitation

## Abstract

**Background:**

Defecating in the open predisposes people to soil transmitted helminthes and diarrhoeal diseases. An estimated 5.6 million Kenyans defecate in the open. Kenya launched a program to eradicate open defecation by 2013 in the rural areas. By end of 2013, only two sub-counties had eliminated open defecation. These are Nambale and Nyando. The study looked at the impact of eradicating open defecation on diarrhea prevalence among children in these two sub-counties.

**Methods:**

Data on diarrhoea morbidity among children under 5 years was extracted from the Kenya Health Information System for all the sub-counties in Busia and Kisumu counties for 2012, 2013 and 2014 respectively. Prevalence was calculated for each sub-county in Kisumu for comparison with Nyando’s. Prevalence was also calculated for each sub-county in Busia County and compared to that of Nambale sub-county. A Mann–Whitney *U* Test was done to test the null hypothesis that diarrhoea prevalence was similar in both open defecation and open defecation free sub-counties.

**Results:**

A Mann–Whitney *U* Test revealed significant difference in diarrhoeal prevalence of open defecation sub-counties (*Md* = 18.4, *n* = 34) and open defecation free sub-counties (*Md* = 9.8, *n* = 5), *U* = 9, *z* = −3.2, *p* = .001. Among the two Counties, Nambale had the lowest prevalence. It recorded a decline from 9.8 to 5.7 % across the three years. Prevalence for diarrhoea cases in Nyando declined from 19.1 to 15.2 % across the three years. Nyando initially had the second highest prevalence in Kisumu County and by 2014 it had the lowest prevalence.

**Conclusions:**

The two sub-counties with open defecation free status had lower prevalence of diarrhoea cases compared to sub-counties which were yet to attain open defecation free status. This suggests that elimination of open defecation may reduce the number of diarrhoea cases.

## Background

Open defecation (OD) is defined as defecation in the fields, bushes, and bodies of water or other open spaces. Over a billion people worldwide practice open defecation [1]. In sub-Saharan Africa, an estimated 215 million people engage in open defecation [2]. Only 3 countries i.e. Ethiopia, Angola and Sao Tome and Principe decreased open defecation by 10 % or more between 2005 and 2010. Only Angola is on track to end open defecation by 2015 [2]. An estimated 5.6 million Kenyans defecate in the open [3]. Open defecation predisposes water and food to faecal contamination and may cause diarrhoea and other faecal-oral diseases. In Kenya, approximately 17,100 children under 5 years die each year from diarrhoea, with 90 % of these deaths being attributed to poor water, sanitation and hygiene [3]. Open defecation may also be a risk factor for soil transmitted helminth infections e.g. hookworm, ascariasis and trichuriasis. Open defecation also causes environmental enteropathy which is a sub-clinical disorder characterized by poor nutrient absorption in the gut and associated with stunting in children [4]. An association has been demonstrated between open defecation and stunting in India, with a 10 % increase in open defecation leading to a 0.7 % increase in both stunting and severe stunting [5]. Open defecation is estimated to account for 54 % of variation in average child height among poor and middle income countries, and 65 % when the population density of open defecation is considered [6].

Economic costs of OD practices are contained within the greater estimates of those with poor sanitation. Poor sanitation costs Kenya US$ 324 million with OD estimated to cost US$ 88 million per year [3]. Out of these costs, US$ 244 million is as a result of premature deaths due to diarrhoea and other sanitation related diseases. Expenditure on healthcare including hospitalization is estimated to cost US$ 51 million. Another US$ 26 million is lost productivity due to time spent looking for a place to defecate. A person practicing OD is estimated to spend 2.5 days per year looking for a private location to defecate [3]. Women are more disadvantaged as they also have to look for a private location to urinate as well as accompany young children to defecate [3]. In Kenya, poor are more likely to practice OD with the poorest quintile being 270 times likely to practice OD than the richest [3]. There are also social costs of OD. These include loss of dignity and privacy with the vulnerable sections of the population more affected. This includes the disabled, women and school going girls. Women may be predisposed to gender based violence as they go in search of sanitary facilities especially at night.

Systematic reviews have shown that improving sanitation can reduce diarrhoea diseases by 32–37 % [7–9]. Kenya launched the open defecation free rural Kenya campaign in 2011. The overall goal was to eradicate open defecation from rural Kenya by end of 2013 [10]. This intervention was spearheaded by the division of environmental health in the ministry of health as well as key non-governmental organizations. The Community- Led Total Sanitation (CLTS) approach was adopted in a bid to eradicate open defecation [11]. Community- Led Total Sanitation (CLTS) was pioneered in Bangladesh in 2000 by Dr Kamal Kar and introduced in Kenya in 2007. It is a participatory methodology for mobilising communities to eliminate open defecation. Communities are facilitated to conduct their own appraisal and analysis of open defecation and take action to become open defecation free. Analysis and appraisal includes mapping areas of open defecation, calculating the amount of faeces produced, medical costs as a result of faecal oral disease burden as well as evoking disgust among the community members due to open defecation. Community-Led Total Sanitation does not provide subsidies and encourages innovation and appropriate local solutions leading to sustainability. A village is declared open defecation free (ODF) once all community members are using latrines and there is no trace of faeces in the environment as verified by a third party. In Kenya, there are three criteria for ODF verification [12]. The first is a complete absence of exposed human faeces within the community. Secondly all households should have access to a latrine which should not predispose users to faecal-oral diseases. Each homestead should have a functional latrine. The squat hole should be covered, the floor should be free of faeces and urine; and the superstructure should provide some privacy. Thirdly all households should have hand washing facility near the latrine. This should be in use and there should be evidence of soap/ash and water. Prior to certification, there is verification of ODF claims by the health workers and village sanitation committee. This is to ensure the village is indeed ODF before external parties can be invited to do ODF certification. In Kenya, certification was initially done by selected non-governmental organizations, the key one being KWAHO (Kenya Water for Health Organization). Currently, there is a move to train local certifiers within each sub-county. This is because certification used to be delayed to due limited capacity of third party certifiers. A typical certification process comprises of three steps. The first being desk study and planning [12]. This includes review of progress reports, forming a certification team and determining sample size. Next is the field visit to collect data. This will involve key informant interviews with natural leaders, government officials, household visits and focus group discussions with community members e.g. women [12]. The certifiers usually take transect walks e.g. through open fields, near water bodies looking for signs of open defecation. Children are also interviewed on where they defecate to corroborate information provided by adults. Each household visited is inspected for a functional latrine with a footpath leading to it to confirm usage. Each latrine is inspected for presence of a drop hole cover, absence of faecal matter on the floor and hand-washing facilities which should show evidence of recent use. The certifiers also verify claims of latrine sharing by households. Lastly there is the data analysis and writing of the certification report which is shared with relevant stakeholders. If a village is certified ODF, then celebrations can be planned [12].

Once certified ODF, the village may celebrate the achievement and erect a signboard or banner detailing the ODF attainment. Celebrations are held at least three months after ODF certification. This is to ensure there is real change before celebration takes place. Prior to celebration, another visit is carried out by third party certifiers. The village sanitation committee continues to be vigilant to ensure ODF is sustained and there is no reversal to open defecation. Attainment of ODF status may be followed by improvement of existing sanitation facilities e.g. through sanitation marketing. As of 2014, CLTS had been implemented in 15 % of villages in rural Kenya and 7 % of villages declared ODF [13]. Counties with the highest number of villages declared ODF were Busia, Kisumu and Siaya. In these counties, 33, 30 and 29 % of villages respectively had been declared ODF [13]. Sanitation and health services in Kenya were devolved after the formation of 47 county governments in 2013. A county is normally divided into sub-counties with the entire country having 265 sub-counties. Out of these only two have been certified ODF. These are Nambale and Nyando. The latter was certified in October 2013 and the former in June 2012 [13]. These are located in Busia and Kisumu counties respectively. The study sought to explore the impact of being declared open defecation free on reported diarrhoea morbidity among infants in these two sub-counties.

## Methods

### Study area

Busia County is located in western Kenya and covers an approximate area of 1695 km^2^. It borders Lake Victoria to the south west, the Republic of Uganda to the west. It has a population of 743,946 according to the 2009 Kenyan population census. It comprises of seven sub-counties. These are Teso North, Teso South, Nambale, Matayos, Butula, Bunyala and Samia [14]. Kisumu County is also located in western Kenya and it covers a total land area of 2,009.5 km^2^ and another 567 km^2^ covered by water. It has seven sub-counties namely: Kisumu East, Kisumu West, Kisumu Central, Nyando, Seme, Nyakach and Muhoroni [Table 1]. It had a population of 968,909 people according to the 2009 national census. It has an average population density of 482 persons per square kilometer. [15].

**Table 1:** Background Characteristics of Kisumu County

Name of Sub-County	Population	Population density (persons/km^2^)	Proportion with access to improved sanitation (%)	Proportion with access to improved water sources (%)	% with primary education
Kisumu East	150,124	1105	65.2	60.1	54.9
Kisumu West	131,246	616	50.9	50.8	59.7
Kisumu Central	168,892	5,165	76.9	71.8	41.7
Seme	98,805	519	34.6	33.3	64.1
Nyakach	133,041	372	62.8	42.2	61.1
Muhoroni	145,764	218	44.4	45.8	61.4
Nyando	141,037	341	52.8	60.6	61.3
Kisumu County	968,909	482	57 %	54 %	57 %

Source: Kenya National Bureau of Statistics, 2013

The study analysed data on diarhoea morbidity for three years in all the sub-counties in Busia and Kisumu counties. Data on diarrhoeal morbidity was accessed from the Kenya Health Information System [16]. Kenya was the first country in sub-Saharan Africa to roll out a completely online national health information system in 2011 [17]. Across the country, health information officers enter data on a wide range of health issues including outpatient and inpatient workloads. Reporting rates are fairly high estimated to be above 90 % [17]. The study utilized two data sets. One was the population estimates. This provides the population estimates for the under 5 year’s cohort in each sub-county per specific year as generated by the Kenya National Bureau of Statistics. The other data set was the outpatient summary for children under 5 years old, commonly called MOH 705 A. This reporting tool captures all outpatient cases seen right from the lowest health facilities i.e. dispensaries up to the highest facilities i.e. county and national referral hospitals. As clinicians see patients, they indicate on tally sheets the number treated per condition. Number of cases per disease seen are tallied daily and also at the end of the month to generate a monthly report. Quality assurance of this data is normally done by the health records and information officers. They are the ones who have rights to enter data as per their area of jurisdiction. Sub-county health management teams normally carry out supportive supervision in all the health facilities. These incorporate on the job training on data quality. Each sub-county normally holds a quarterly data review meeting convened by the sub-county health information and records officer. These are normally attended by officer in charge of health facilities. Any gaps in data quality are identified and addressed.

Each annual report was downloaded in the form of excel sheets and annual diarrhoea morbidity extracted for each sub-county. The study utilized data for 3 years for all the sub-counties in Busia and Kisumu counties. This was from 2012 to 2014, with each year starting from January to December. Prevalence of diarrhoea was determined for each sub-county across the three years. A Mann–Whitney U Test was done to test the null hypothesis that diarrhoea prevalence was similar in both open defecation and open defecation free sub-counties. Ethical approval was not necessary as the study utilized data sets available on a public website. The data on the Kenya Health Information System is normally de-identified to protect patient confidentiality and privacy.

## Results

In Busia County, only two sub-counties recorded a decline in prevalence of diarrhoea cases across the three years. These are Nambale and Bunyala (Table 2). This was from 9.8 to 5.7 % for Nambale and 38.6 to 31.6 % for Bunyala. Teso South, Butula and Samia sub-counties initially recorded a decline followed by an increase in prevalence of diarrhoea cases across the three years. Teso North sub-county recorded an increase in prevalence of diarrhoea cases across the three years. Nambale had the lowest prevalence of diarrhoea cases in Busia County across the three years.

**Table 2:** Background Characteristics of Busia County

Name of Sub-County	Population	Population density (persons/km^2^)	Proportion with access to improved sanitation (%)	Proportion with access to improved water sources (%)	% with primary education
Nambale	94,637	398	50.7	72.6	63.3
Teso South	137,924	460	78.6	50.6	60.9
Butula	121,870	493	52.4	72.8	62.9
Matayos	111,345	568	68.7	71.6	60.8
Teso North	117,947	452	57.3	46	61.9
Bunyala	66,723	354	29.4	55.7	60.5
Samia	93,500	353	77.2	61.8	62.2
Busia County	743,946	437	61 %	61 %	62 %

Source: Kenya National Bureau of Statistics, 2013

**Table 3:** Prevalence of Diarrhoea among Infants in 2012, 2013 and 2014 for sub-Counties in Busia and Kisumu Counties

Name of Sub-County	Prevalence of Diarrhoea 2012	Open Defecation Status 2012	Prevalence of Diarrhoea 2013	Open Defecation status 2013	Prevalence of Diarrhoea 2014	Open Defecation Status 2014
Kisumu West	17.3	OD	13.9	OD	26.4	OD
Kisumu East	15.3	OD	17.2	OD	22.3	OD
Seme	19.9	OD	18.7	OD	32.9	OD
Nyakach	16.7	OD	18.6	OD	19.2	OD
Muhoroni	16.3	OD	16.8	OD	19.5	OD
Nyando	19.1	OD	15.6	ODF	15.2	ODF
Kisumu County	20.1		16.8		21.9	
Nambale	9.8	ODF	7.2	ODF	5.7	ODF
Teso South	15.4	OD	12.1	OD	17.2	OD
Butula	18.8	OD	15.4	OD	19.1	OD
Matayos	17.5	OD	21.2	OD	27.1	OD
Teso North	14.7	OD	16.9	OD	22.7	OD
Bunyala	38.6	OD	35.7	OD	31.5	OD
Samia	17.5	OD	16.8	OD	18.1	OD
Busia County	17.8		16.8		19.6	

*OD* Open Defecation, *ODF* Open Defecation Free

In Kisumu County, the prevalence of diarrhoea cases in Nyando declined from 19.1 to 15.2 % across the three years. Muhoroni, Kisumu West and Seme sub-counties initially recorded a decline followed by an increase in prevalence of diarrhoea cases across the three years. Kisumu East, Nyakach and Muhoroni sub-counties recorded an increase in prevalence of diarrhoea cases across the three years. In 2012, Kisumu East had the lowest prevalence of diarrhoea cases, and Nyando had the second highest prevalence. In 2013, Kisumu West had the lowest prevalence of diarrhoea cases. In 2014, Nyando had the lowest incidence proportion of diarrhoea cases (Table 3). A Mann–Whitney U Test revealed significant difference in diarrhoeal prevalence of open defecation sub-counties (*Md* = 18.4, *n* = 34) and open defecation free sub-counties (*Md* = 9.8, *n* = 5), *U* = 9, *z* = −3.2, p = .001*, r* = .5.

## Discussion

This study shows some promising results that eradicating open defecation coupled with practice of hygiene may reduce diarrhoea in children. The two sub-counties certified ODF showed a decline in diarrhoea cases in children across the three years compared to sub-counties yet to attain ODF status. Nambale was declared ODF in May 2012. It had the least prevalence of diarrhoea cases across the three years in Busia County. Nyando was declared ODF in October 2013 and by 2014 it had the least prevalence of diarrhoea cases in Kisumu County. The first randomised control trial on effectiveness of CLTS was done in Mali [18]. It found no difference in diarrhoeal prevalence among children in CLTS and control villages. Though there was a significant difference in reduction in the prevalence of bloody diarrhoea. The risk of loose or watery stool was reduced by 24 % among children not being breastfed exclusively. Diarrhoea related mortality in children under- 5 years was significantly lower in CLTS villages. Children under- 5 years in CLTS villages were taller and less likely to be stunted [18]. The authors concluded that CLTS prevented diarrhoea and reduced environmental enteropathy through reduced environmental faecal contamination and possible improvements in hand hygiene behaviours. In the Mali study, 97 % of villages were declared ODF. Follow up found some villages had reverted to open defecation as human faeces in latrine floor or compound was observed in 10 and 5.4 % of CLTS households. Also over a third of CLTS households shared latrines. In Kenya, certification guidelines state that each household should have its own latrine and ODF villages should have close to 100 % latrine coverage [12]. Health outcomes in the Mali study were measured one and a half year after being declared ODF and two years after implementing CLTS unlike Nambale. In Nambale, CLTS began to be implemented in 2009 with the first village declared ODF in 2009. In May 2012 all the 179 villages in Nambale became ODF and this feat was marked during the 2012 world toilet day celebration held in Nambale [19]. This means that ODF may have been attained earlier and the public health staff did rigorous follow up before third party certifiers were brought in. A study found that in Nambale the number of mean monthly diarrhoeal cases declined after elimination of open defecation. These reduced by 28.4 % after the first year and 38.3 % after the second year of becoming ODF [19]. Nyando presents a different scenario as it was declared ODF in 2013. In 2012, it had the second highest prevalence. In 2013, it had the second least prevalence and the following year it had the least prevalence in Kisumu County. This may indicate a gradual improvement as the process of attaining ODF status was implemented. Whereas CLTS is the national strategy, other forms of sanitation are implemented in these two counties by non-governmental organizations and the government. These include subsidy based approaches like construction of toilets for public use e.g. in schools; and for emergency situations e.g. people affected by disasters.

A study in Ethiopia found that latrine coverage was 79.4 % in CLTS villages compared to 59.1 % in non-CLTS villages. Two weeks prevalence of diarrhoea was the same in both CLTS and non- CLTS villages [20]. In this scenario, the CLTS villages were yet to attain ODF status and this may explain the similar diarrhoea prevalence. In Philippines a study compared prevalence of soil transmitted helminthes in two villages where CLTS had been implemented attaining open defecation status. These were compared to two non- CLTS villages. One CLTS village had a significantly higher cumulative prevalence of soil transmitted helminthes at 67.4 % while the other had a significantly lower cumulative prevalence of 4.9 %. On the other hand, the non- CLTS villages had cumulative prevalence of 16.7 and 16.8 %. Reasons given for the high prevalence in one CLTS village include possible reversion to open defecation and non-utilization of latrines [21].

This study could be having some limitations. Not all diarrhoea cases are managed at the health facility in Kenya. Some are managed at the household level and community level by caregivers and community health volunteers. In western Kenya, a study found that healthcare was sought outside the home for 77.8 % of children with diarrhoea. Places visited as first source of care included licensed (62 %) and unlicensed (11 %) providers and pharmacies (27 %) [22]. According to the Kenya demographic and health survey 2014, 58 % of children with diarrhoea two weeks preceding the survey had been taken to a healthcare provider for treatment. In Kisumu and Busia counties the figure was 59.5 and 55.5 % [23]. Diarrhoea cases are also be influenced by meteorological factors and this may not be constant across the sub-counties. Though comparing sub-counties within the same county may reduce some of these variations e.g. in Kisumu county Nyando, Nyakach and Muhoroni sub-counties are prone to flooding [15]. Another limitation is ecological fallacy, which infers association at the population level whereas none may exist at the individual level. Data collected through the routine health information system is also liable to missing data. As from July 2014, Kenya introduced Rota virus vaccine in all its public health facilities. The study cannot ascertain whether coverage was similar across all sub-counties for the six months in 2014.

## Conclusions

These two sub-counties with open defecation free status recorded a decline in diarrhoea morbidity among children compared to sub-counties yet to attain open defecation status. This suggests that eliminating open defecation may reduce diarrhoea cases.

## Abbreviations

CLTS, Community- led total sanitation; OD, open defecation; ODF, open defecation free
